# Rapid Pathogen Purge by Photosensitive Arginine–Riboflavin Carbon Dots without Toxicity

**DOI:** 10.3390/ma16196512

**Published:** 2023-09-30

**Authors:** Selin S. Suner, Venkat R. Bhethanabotla, Ramesh S. Ayyala, Nurettin Sahiner

**Affiliations:** 1 Department of Chemistry, Faculty of Sciences & Arts, and Nanoscience and Technology Research and Application Center (NANORAC), Canakkale Onsekiz Mart University Terzioglu Campus, Canakkale 17100, Turkey; sagbasselin@gmail.com; 2 Department of Chemical, Biological, and Materials Engineering, Materials Science and Engineering Program, University of South Florida, Tampa, FL 33620, USA; bhethana@usf.edu; 3 Department of Ophthalmology, Morsani College of Medicine, University of South Florida Eye Institute,12901 Bruce B Down Blvd, MDC 21, Tampa, FL 33612, USA; rsayyala@gmail.com

**Keywords:** dual-fluorescence carbon dots (CQ dots), arginine amino acid-derived CQ dots, fluorescein sodium salt/riboflavin dye, antimicrobial, photodynamic therapy, biocompatible CQ dots

## Abstract

Photo-activatable antipathogenic carbon dots (CDs) were prepared by carbonization of citric acid and arginine (Arg) via 3 min microwave treatment for use in the eradication of common microorganisms. Nitrogen-doped Arg CDs were spherical in shape with a size range of 0.5 to 5 nm. The Arg CDs were modified with fluorescent dyes, such as fluorescein sodium salt (FSS, as Arg-FSS) and riboflavin (RBF, as Arg-RBF), to improve antimicrobial potency by enhancing their application in photodynamic therapy. The modified Arg CDs afforded fluorescence emission properties at 520 nm in the green region in addition to excellent blue fluorescence intensity at 420 nm under 345 nm excitation upon their FSS and RBF conjugation, respectively. Although the cytotoxicity of Arg CDs was decreased for Arg-RBF CDs to 91.2 ± 0.7% cell viability for fibroblasts, the Arg-based CDs could be safely used for intravenous applications at 1000 μg/mL concentration. The Arg CDs showed broad-spectrum antimicrobial activity against common pathogens and the minimum inhibitory concentration of Arg CDs was almost two-fold decreased for the modified forms without UV light. However, faster and more effective antibacterial activity was determined for photosensitive Arg-RBF CDs, with total bacterial eradication upon UV-A light exposure for 30 min.

## 1. Introduction

Photo-activated materials, including titanium dioxide [1], zinc oxide [2], graphitic carbon nitride [3], some photosensitive dyes [4], and fluorescence carbon dots [5,6], induce the photochemical generation of free radicals or singlet oxygen, which are reactive oxygen species (ROS) upon ultraviolet (UV) light treatment. This ROS production by the photodynamic process induces cytotoxicity against microorganisms via oxidative damage and provides rapid microbial inhibition or eradication [7]. Carbon dots (CDs) are green, non-toxic, biocompatible nanomaterials [8] that are ecofriendly [9] and inexpensive to synthesize in contrast to most light-sensitive antimicrobial materials [5,10,11]. Furthermore, these materials can pass through the cell membrane because of their size, e.g., a maximum size distribution of about 20 nm [12,13]. Positively charged CDs were designed as nano-antibiotics, providing great antimicrobial ability without significant toxicity [14]. The great fluorescence intensity of CDs makes them excellent antimicrobial materials under light treatment with their light-activated reactive oxygen species (ROS) production property inducing toxicity against pathogenic organisms. The produced ROS species by photosensitive CDs oxidize some significant biomacromolecules, including proteins, nucleic acids, and lipids, engendering cytotoxicity and cell wall damage to pathogenic organisms [15,16,17,18]. The two main bacterial/fungal inhibition mechanisms supported by the literature are DNA and membrane damage by the release of ROS from photoinduced CDs. Therefore, a wide spectrum of microorganisms could be eradicated by employing CDs via photodynamic therapy applications because of the non-specific killing mechanism [19].

N-doped Arg CDs were previously synthesized with a citric acid and arginine mixture by employing a microwave synthesis method in a short time, which was demonstrated as a photodynamic antibacterial material after modification with amine group-containing agents [20], and effective antibacterial composite forms were reported without light activation [21]. However, these modifying amine group-containing antimicrobial agents and their metal nanoparticle-containing composites caused toxic effects, limiting the use of the Arg CDs for in vivo applications. In this study, photoinducible antibacterial Arg CDs by modification with dyes such as riboflavin (RBF) and fluorescein sodium salt (FSS) without any toxicity are reported. Riboflavin (RBF) is an essential B_2_ vitamin that plays a significant role in energy metabolism, fatty acid oxidation, the Krebs cycle, and purine catabolism [22]. Also, some important coenzymes in metabolism, such as flavin mononucleotide and flavin adenine dinucleotide, which function in electron transport during various redox reactions in the energy generation cycle, are produced from the RBF molecule. In the human diet, 1.4 mg RBF is recommended per day and its deficiency can cause dermatitis and keratosis-type skin disorders, nervous system problems, digestive troubles [23], as well as migraines, childhood neuropathy, anaemia, cataracts, oxidative stress, diabetes mellitus, hypertension, and cancer [22]. Furthermore, some pathogens, including adenovirus, HIV virus, parvovirus, Gram-positive and Gram-negative bacteria, and *Leishmania* protozoa, can be inactivated by RBF under UV light without any toxicity [24,25,26]. In particular, photo-induced RBF with UV light exposure could inactivate DNA and RNA viruses because of photosensitivity of RBF via oxidation of DNA and RNA [4]; therefore, it could be used in the inactivation of viruses for vaccine design or virus-based gene therapy applications [22]. Riboflavin (RBF) and fluorescein sodium salt (FSS) are generally preferred in bioimaging as diagnostic agents in clinically administered ophthalmic applications because of their fluorescence property in addition to non-toxic effects on the ocular system [27,28]. Panda et al. reported that RBF is a promising molecule in the treatment of corneal ulcers via its photo-activated effect under UV-A irradiation [29]. In a different study, it was shown that FSS did not possess antibacterial ability, unlike riboflavin [28], but was considered to be a safe agent for in vivo diagnostic applications [30]. Therefore, the photodynamic antimicrobial activity of N-doped Arg CDs could be improved by modification with RBF and FSS fluorescence agents as benign materials for biomedical use. In this study, modified CDs, such as Arg-FSS and Arg-RBF CDs, were prepared via *N*,*N*’-carbonyldiimidazole (CDI) as the coupling agent in the reaction of Arg CDs with the fluorescence dyes. The optical properties of the Arg, Arg-FSS, and Arg-RBF CDs were evaluated using UV-Vis and fluorescence spectroscopies. The toxicity of these materials was determined by cytotoxicity tests against L929 fibroblast cells and hemocompatibility tests according to hemolysis and blood clotting analyses. Moreover, the antimicrobial susceptibility of Arg, Arg-FSS, and Arg-RBF CDs were evaluated against a wide range of pathogenic microorganisms, including Gram-negative *Escherichia coli* (*E. coli*, ATCC 8739) and *Klebsiella pneumoniae (K. pneumoniae*, ATCC 700603), Gram-positive *Staphylococcus aureus* (*S. aureus*, ATCC 6538) and *Bacillus subtilis (B. subtilis*, ATCC 6633), and *Candida albicans* (*C. albicans*, ATCC 10231) yeast utilizing a microtiter assay to find the minimum inhibitory concentration (MIC). In addition, photodynamic bacterial inhibition of *B. subtilis* in the presence of the prepared CDs was investigated under dark conditions and under UV-A light-mediated exposure to show the photo-induced antipathogenic activity of these materials.

## 2. Materials and Methods

### 2.1. Materials

L-Arginine (Arg, >98%, Sigma-Aldrich, St. Louis, MO, USA) and citric acid monohydrate (CA, >99%, Carlo Erba, Emmendingen, Germany) were used in the preparation of carbon dots. In the conjugation reaction, fluorescein sodium salt (FSS, >95%, Sigma-Aldrich, USA) and riboflavin (RBF, >99%, Sigma Aldrich, USA) as fluorescence dyes and 1,1′-carbonyldiimidazole (CDI, >97%, Sigma Aldrich, Darmstadt, Germany) as a coupling agent were used. The L929 fibroblast cell line (mouse C3/An connective tissue, SAP Institute, Ankara, Turkey) was used as a model cell line for toxicity analysis. Dulbecco’s Modified Eagle’s Medium (DMEM, L-glutamine, 15 mM HEPES, 1.2 g/L NaHCO_3_), fetal bovine serum (FBS), trypsin-EDTA (0.25%), and antibiotic (10,000 U/mL penicillin, 10,000 μg/mL streptomycin) were obtained from Pan Biontech GmbH (Aidenbach, Germany). Trypan Blue (0.5% solution, Biological Industries, Kibbutz Beit-Haemek, Israel), thiazolyl blue tetrazolium bromide (MTT agent, BioFroxx, Einhausen, Germany), and dimethyl sulfoxide (DMSO, 99.9%, Carlo-Erba) were used as received. As model microorganisms, *Escherichia coli* ATCC 8739, *Klepsiella pneumoniae* ATTC 700603, *Staphylococcus aureus* ATCC 6538, *Bacillus subtilis* ATCC 6633, and *Candida albicans* ATCC 10231 were purchased in KWIK-STIK format (Microbiologics, France). Nutrient agar (NA, Condolab, Madrid, Spain) and nutrient broth (NB, Merck, Darmstadt, Germany) were used as received. Acetone (96%, Birkim, Gungoren, Turkey) and ethanol (98%, BRK, Turkey) were obtained from a local vender. Aqueous solutions were prepared with ultrapure water of about 18.2 M·Ω·cm using a Millipore-Direct Q UV3 (Burlington, MA, USA). 

### 2.2. Synthesis of Arg CDs and Conjugation of Arg CDs with Fluorescein Sodium Salt and Riboflavin

A microwave-assisted preparation technique was used for the synthesis of Arg CDs in a single step by reacting citric acid and arginine, as reported by Suner et al. [21]. Briefly, a 3:1 weight ratio of CA:Arg solution was prepared in 3 mL of DI water at 400 mg/mL concentration. After mixing for 30 min at 200 rpm, this solution was carbonized using a domestic microwave at 1000 W (Beko, Manisa, Turkey) for 3 min. The synthesized Arg CDs were dispersed in 1 mL deionized water through sonication for 5 sec and then filtered through a 0.22 µm pore filter. To remove unreacted chemicals, Arg CD suspension was placed in a dialysis membrane and this enclosed membrane was placed in an excess amount of DI water. Every 30 min, DI water was replaced with fresh water and the washing process was continued for 4 h. The washed Arg CDs were precipitated in an excess amount of acetone. Then, the supernatant was decanted, and the precipitate was suspended in 30 mL of deionized water. Next, Arg CD suspension in water was centrifuged at 10,000 rpm for 10 min and this process was repeated three times to remove water from the Arg CDs. The washed Arg CDs were dried in a 50 °C oven for 24 h and stored in a clean centrifuge tube. 

In the conjugation reaction of Arg CDs with the fluorescence dyes, 2.5 mg/mL concentration of 4 mL FSS solution, adjusted to pH 12 with 0.2 M NaOH solution, was reacted with the same molar amount of 1.2 mg/mL CDI solution in 4 mL of deionized water at 80 °C for 2 h. Then, 200 mg Arg CDs was added to the reaction and stirred at the same conditions for 24 h at 80 °C to prepare Arg-FSS CDs. Similarly, the Arg-RBF CDs were prepared by the reaction of 200 mg Arg CDs with 4 mL of 1.2 mg/mL CDI aqueous solution at 80 °C for 2 h. Subsequently, 4 mL of 2.83 mg/mL RBF solution, adjusted to pH 12 with 0.2 M NaOH solution, was added dropwise to the reaction and stirred at the same conditions for 24 h at 80 °C. For precipitation of the modified Arg-FSS and Arg-RBF CDs, the aqueous suspension was added to 200 mL of acetone and then washed twice to remove unreacted chemicals by centrifugation at 10,000 rpm for 10 min. The Arg-FSS and Arg-RBF CDs were dried in a 50 °C oven for 24 h and stored in clean centrifuge tubes. 

### 2.3. Characterization of Arg, Arg-FSS, and Arg-RBF CDs

The size and crystalline structure of Arg CDs were analyzed using a high-resolution transmission electron microscope (HR-TEM, Tecnai TF-20, Hillsboro, OR, USA) at 200 kV imaging, employing lacey carbon support film on 200–300 mesh copper TEM grid. Furthermore, the dynamic light scattering (DLS) of 1000 μg/mL CD suspension in 0.01 mol KNO_3_ aqueous solution was measured using a particle size analyzer (Nanobrook Omni, Brookhaven Instrument, Holtsville, NY, USA) to give the hydrodynamic size distribution of the prepared CDs. The zeta potential of the CD suspension in 0.01 mol KNO_3_ aqueous solution was determined by zeta potential measurement (Zetasizer, NanoZS90, Brookhaven, NY, USA). The chemical composition of Arg CDs and conjugated forms were determined by FT-IR spectroscopy (Thermo, Nicolet-iS10, Waltham, MA, USA) in the 650 to 4000 cm^−1^ range using the ATR technique. Moreover, UV-Vis spectroscopy (T80+UV/Vis spectrometer, PG Instrument, Leicestershirem, UK) and fluorescence spectroscopy (Thermo Scientific, Lumina, Waltham, MA, USA) of Arg-based CD suspensions at 100 μg/mL concentration in DI water were employed to obtain the optical properties of the CDs. Fluorescence measurements were carried out at different excitation wavelengths between 345 and 480 nm in the 350–650 nm emission wavelength range. The quantum yield (QY) values of Arg, Arg-FSS, and Arg-RBF CDs were calculated based on the method described by Sahiner et al., 2019 [31]. Briefly, QY values of the CD suspensions in DI water were evaluated with respect to a quinine standard prepared in 0.5 M aqueous H_2_SO_4_ solution, which had a quantum yield value of 54% at 345 nm excitation. In addition, as a standard for Arg-FSS and Arg-RBF CDs, fluorescein dye was prepared in 0.1 N NaOH and its QY value was determined to be 92% at 520 nm excitation [32]. 

### 2.4. Hemocompatibility of Arg, Arg-FSS, and Arg-RBF CDs

The hemocompatibility of bare and modified Arg CDs was tested by hemolysis and blood clotting analyses. For the hemocompatibility analysis in human blood, ethics committee approval was obtained from the Human Research Ethics Committee of Canakkale Onsekiz Mart University (2011-KAEK-27/2022). Before the analysis, 10 mL of blood was taken from a healthy human and quickly placed in an anticoagulant agent-containing tube. Arg-based CD suspensions at 1000 μg/mL concentration in serum physiologic (SP, 0.9% NaCl solution) were used for the hemolysis and blood clotting analyses. Briefly, 10 mg CDs was suspended in 10 mL of SP and 200 μL of diluted blood at a 1:1.25 ratio of blood:SP solution. The blood- and sample-containing tubes were placed in a shaking water bath under slow shaking at 37 °C for 1 h. As positive and negative controls, 200 μL of diluted blood was suspended in only 10 mL of deionized water and 10 mL of SP, respectively, and incubated under the same conditions. Then, these tubes were centrifuged at 100× *g* for 5 min, and the absorbance of the supernatant solution was measured using a UV-Vis spectrophotometer at 542 nm to evaluate the hemolysis ratio (%) of Arg-based CDs, according to Equation (1). All analysis was performed in triplicate and expressed with ± SD.

Hemolysis ratio (%) = (A_SAMPLE_ – A_NC_)/(A_PC_ – A_NC_) × 100 (1)

where A_SAMPLE_ is the absorbance value of the blood solution containing Arg-based CDs; A_NC_ is the absorbance value of the negative control, which is 200 μL of diluted blood suspended in 10 mL of SP; and A_PC_ is the absorbance value of the positive control, which is 200 μL of diluted blood suspended in 10 mL of deionized water.

For the Arg-based CDs blood clotting tests, 100 μL of 10 mg/mL Arg-based CD suspension in SP was added to flat bottom tubes. Separately, 64 μL of 0.2 M CaCl_2_ solution was mixed with 810 μL of blood, and 200 μL of the blood solution was immediately added to the suspension of the CDs in the tube. These tubes were placed in a shaking water bath under slow shaking at 37 °C for 10 min and centrifuged at 100× *g* for 1 min. The supernatant of 10 mL of blood solution was carefully diluted with 40 mL of deionized water. Separately, 250 μL of only blood was diluted with 50 mL of deionized water as the control. After 1 h incubation at 37 °C, the absorbance of the blood solution was measured using a UV-Vis spectrophotometer at 542 nm to determine the blood clotting index (%) of Arg-based CDs according to Equation (2). All analysis was performed in triplicate and expressed with ± SD.

Blood clotting index (%) = (A_SAMPLE_/A_CONTROL_) × 100 (2)

where A_SAMPLE_ is the absorbance value of the blood solution, which interacted with Arg-based CDs. A_CONTROL_ is the absorbance value of 250 μL of only blood suspended in 50 mL of deionized water.

### 2.5. Cytotoxicity of Arg, Arg-FSS, and Arg-RBF CDs

L929 fibroblast cells were employed for the cytotoxicity analysis of Arg CDs. The cells were grown in DMEM + 10% FBS + 1% antibiotic as the culture media and 100 μL of 1 × 10^5^ cell/mL suspension in medium was added to each well of a 96-well plate. The plate was incubated in 5% CO_2_ and 95% air at 37 °C. After 24 h, the medium was removed from the wells and 100 μL of the CD suspension in medium at 50–1000 μg/mL concentrations was placed on the attached fibroblast cells in the wells. The plate was incubated for an additional 24 h. Subsequently, the medium from the wells was removed and the attached cells were washed with PBS three times. For the analysis of cell viability, 100 μL of 0.5 mg/mL fresh MTT solution was placed on the cells in the wells and incubated at 37 °C for 2 h under dark conditions. Then, MTT solution was decanted from the wells and 0.2 mL of DMSO was added. After 10 min, the absorbance value of solution in the wells was measured using a plate reader (Thermo, Multiskan Sky, Waltham, MA, USA) at 590 nm. All analysis was performed in triplicate and expressed with ± SD. Student’s *t*-test in GraphPad Prism software version 7 was utilized to determine statistical differences in the cytotoxicity results compared to the control group. A *p*-value below 0.05 was given as statistically significant.

### 2.6. Antimicrobial Activities of Arg, Arg-FSS, and Arg-RBF CDs

The antibacterial susceptibility of *E. coli*, *K. pneumoniae*, *S. aureus*, and *B. subtilis* and antifungal activity against *C. albicans* were investigated by microtiter assay. Briefly, 50 mg/mL concentration of Arg-based CDs was suspended in SP and sterilized under a UV lamp at 320 nm for a few minutes. In a 96-well plate, 100 μL of liquid growth suspension was added to each well. Then, 100 μL of the prepared CD suspension at 50 mg/mL concentration was added to the first well, which was serially diluted with nutrient broth (NB). Separately, bacterial/fungal suspension was adjusted to McFarland standard 0.5 in NB, and 10 μL of this bacterial/fungal suspension was placed in the wells. The plate was incubated at 37 °C for 18–24 h. The well in which the lowest concentration of CDs exhibited no visible growth was considered to be the minimum inhibitory concentration (MIC). All analysis was performed in triplicate and expressed with ± SD.

### 2.7. Light-Activated Antimicrobial Capacity of Arg, Arg-FSS, and Arg-RBF CDs

The light-activated antibacterial effects of the Arg-based CDs with time upon UV light exposure were investigated against *B. subtilis*. In brief, 1000 μg/mL concentration of Arg-based CD suspension was prepared in 1 mL of SP, which contained bacterial colonies at McFarland 0.5. These suspensions were incubated under dark conditions or UV light exposure (315–400 nm, UV-A, 300 W, Ultra Vitalux, Osram GmbH, Munich, Germany). After 5, 15, and 30 min incubation times under both conditions, 100 µL of suspension was removed, diluted with SP, and inoculated on NA to count the living colonies of bacteria. After incubation at 37 °C for 18–24 h, the bacterial cell viability (%) was evaluated in comparison to the control group. All analysis was performed in triplicate and expressed with ± SD.

## 3. Results and Discussion

Arg CDs were synthesized using arginine (Arg) as a nitrogen source and citric acid as a carbon source based on our earlier report [16,17]. Following our previous work, Arg and citric acid solution at a 3:1 weight ratio was mixed and placed in a microwave for 3 min to prepare Arg CDs. Therefore, a one-step, easy, and inexpensive synthesis procedure was employed herein for the preparation of N-doped Arg CDs. The size distribution of the synthesized CDs was determined by TEM images and DLS measurements.

**Figure 1:**
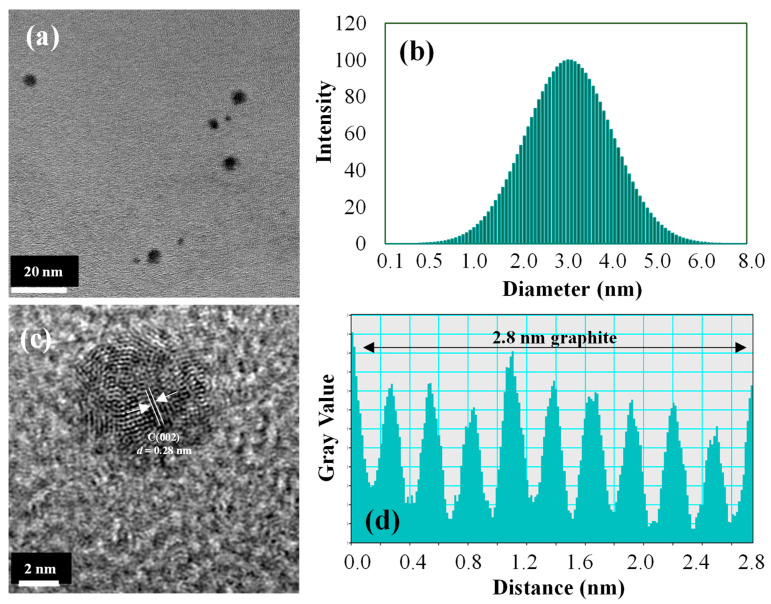
(**a**) TEM image, (**b**) hydrodynamic size distribution, (**c**) HR-TEM image, and (**d**) gray value graphitic plot to show graphitic nature of Arg CDs.

As seen in Figure 1a,b, the Arg CDs were only a few nanometers in size, in the range of 0.5–5 nm. The graphitic network of the CDs was readily observed in high-resolution TEM images, as demonstrated in Figure 1c. The crystalline structure of the CDs was visible in the 0.28 nm lattice space corresponding to graphitic carbon material, as seen in Figure 1d. 

**Figure 2:**
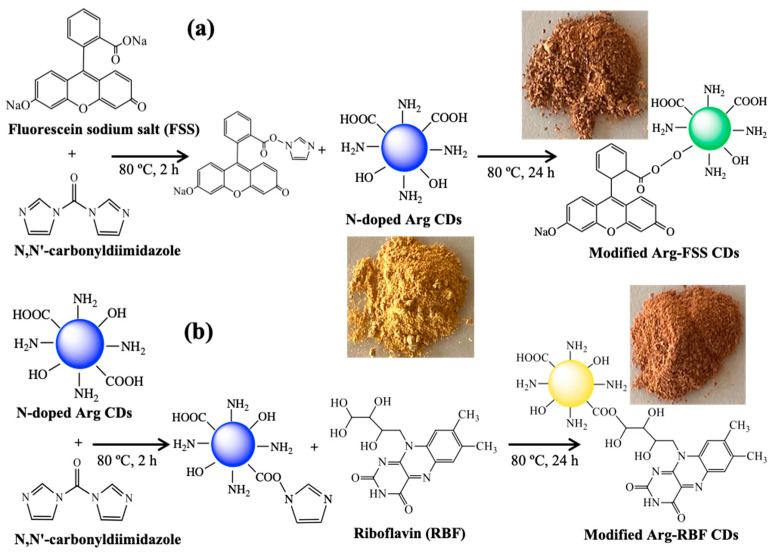
The conjugation reaction mechanisms of Arg CDs with (**a**) fluorescein sodium salt (FSS) and (**b**) riboflavin (RBF) dyes and their corresponding digital camera images.

The Arg CDs were separately modified with fluorescein sodium salt (FSS) and riboflavin (RBF) fluorescence dyes by means of a conjugation reaction, as illustrated in Figure 2. In the conjugation reaction between the dyes and Arg CDS, *N,N’*-carbonyldiimidazole (CDI) was utilized as the coupling agent. As depicted in Figure 2a, the carboxylic acid groups of FSS could react with CDI, and then the hydroxyl groups of Arg CDs could bind to the carboxylic acid groups of FSS through the CDI coupling agent. In other words, the carboxylic acid groups of Arg CDs were first activated with CDI during a 2 h reaction and then reacted with the hydroxyl groups of RBF for 24 h, as shown in Figure 2b.

It is apparent that CDI coupled the hydroxyl and carboxylic acid groups in the conjugation reactions between the dyes and Arg CDs via opposite reactions because of the utilization of different functional groups for the FSS and RBF dyes, which had carboxylic acid and hydroxyl groups, respectively. As seen in the digital camera images in Figure 2, the color of Arg CDs changed from light yellow to brick red of slightly different intensities upon the conjugation reaction with FSS and RBF dyes. These images indicated that modified Arg-FSS and Arg-RBF CDs were successfully attained by the two-step reactions. 

**Figure 3:**
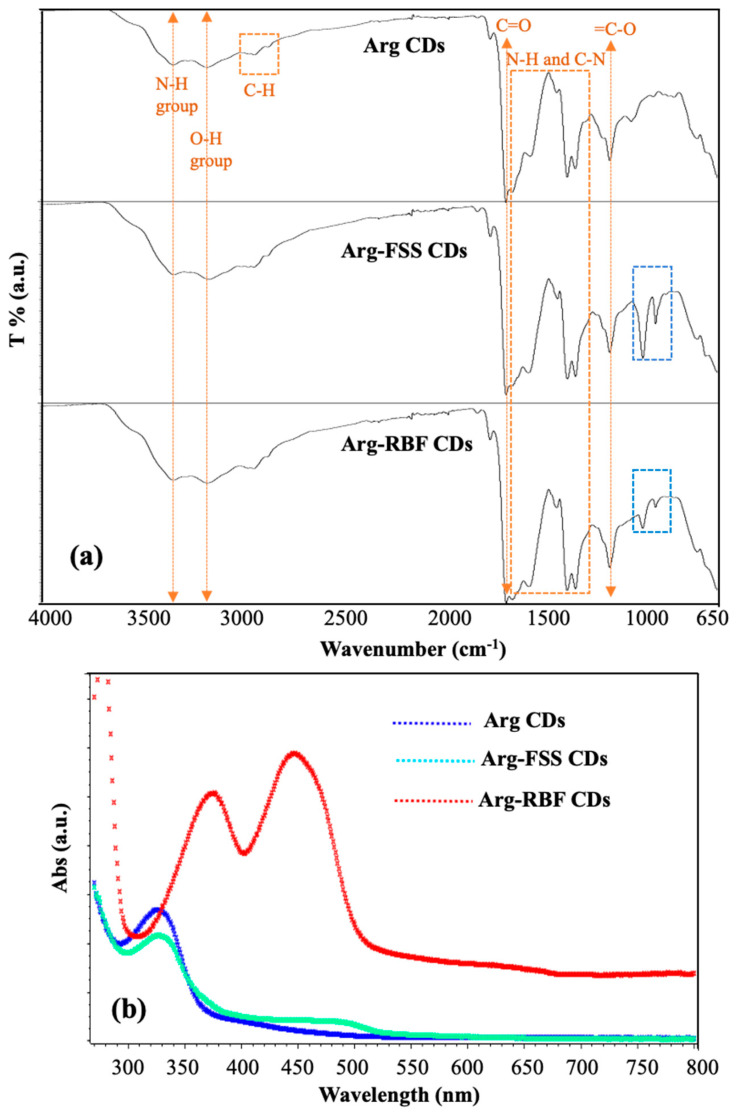
(**a**) FT-IR spectra and (**b**) UV-Vis absorption spectra of Arg, Arg-FSS, and Arg-RBF CDs.

The functional groups of the prepared CDs were assessed by FT-IR spectroscopic analysis and the spectra are shown in Figure 3a and indicated by yellow arrows, yellow and blue squares ow the corresponding functional groups stretching and vibration. The absorption peaks at 3360, 3185, 2980, and 1695 cm^−1^ were assigned to the N-H, O-H, C-H, and C=O groups, respectively, belonging to Arg CDs. The bands between 1650 and 1360 cm^−1^ were attributed to the NH and CN groups of the N-doped Arg-based CDs. Furthermore, the peak at 1225 cm^−1^ belonged to the vibration of the =C-O group present in Arg-based CDs [21]. Specifically, dye-conjugated Arg-FSS and Arg-RBF CDs also showed the same absorption bands at approximately 1020 and 950 cm^−1^ originating from the aromatic C-H in-plane band and aryl ether bonds, respectively [33]. 

The optical properties of the modified Arg-FSS and Arg-RBF CDs were investigated by means of UV-Vis and fluorescence spectroscopies and compared with those of bare Arg CDs. As reported earlier, the absorption peak at λmax 320 nm was assigned to the n-π* transition of surface groups such as carbonyl groups and the nitrogen-rich surface [20,21]. As shown in Figure 3b, this strong band was observed in Arg CDs and the modified form with FSS. This peak at 320 nm disappeared in modified Arg-RBF CDs because of the conjugation reaction between the hydroxyl groups of RBF and the C=O groups of Arg CDs. The specific absorption band of FSS, which was obtained at 494 nm, was observed due to the FSS group in the Arg-FSS CDs [34]. Similarly, the highest peaks at 374 and 445 nm were attributed to characteristic peaks of RBF in the Arg-RBF CDs [35]. 

**Figure 4:**
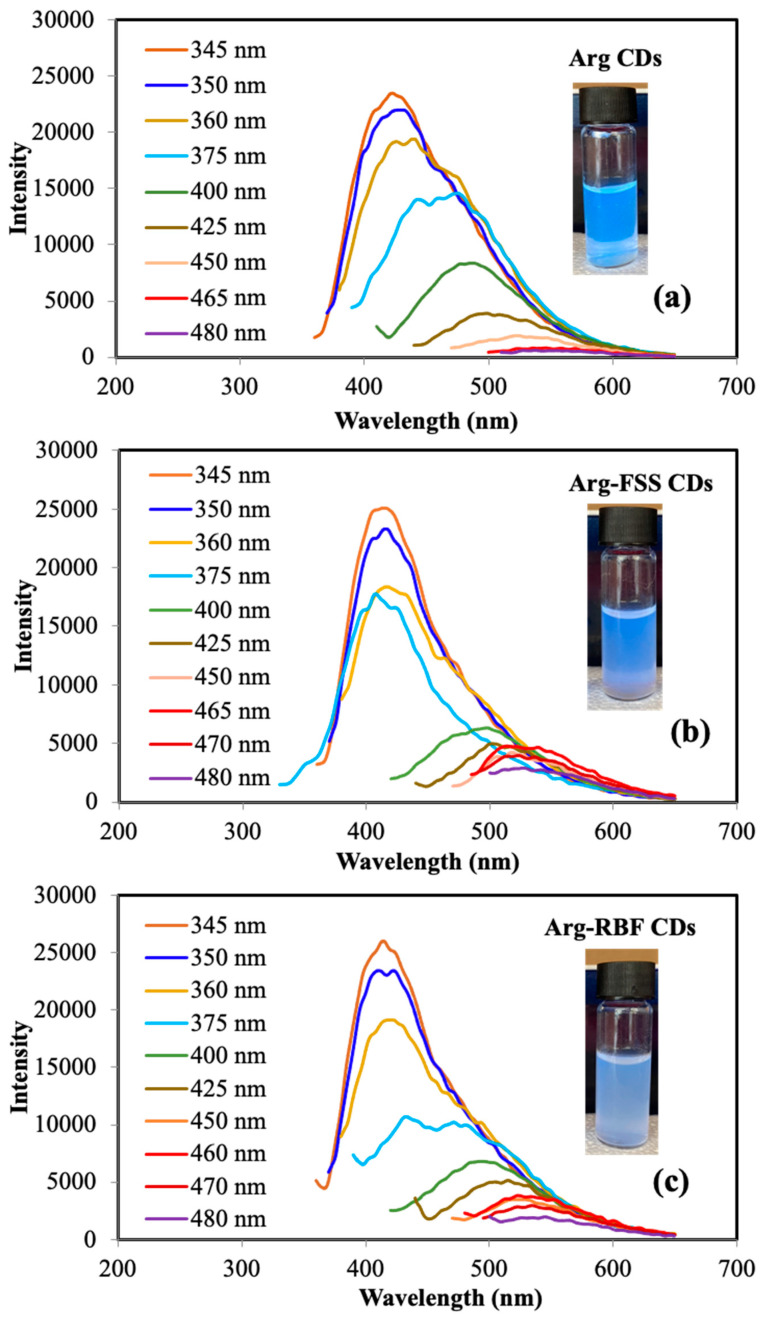
Fluorescence emission spectra of (**a**) Arg, (**b**) Arg-FSS, and (**c**) Arg-RBF CDs at different excitation wavelengths in 345–480 nm range and their digital camera images.

The fluorescence emission spectra of the CDs in the range of 345–480 nm excitation wavelengths are shown in Figure 4a–c, respectively. 

As seen in Figure 4a, the emission wavelength of bare Arg CDs was shifted to the green-yellow area from the purple-blue region by increasing the excitation wavelength between 345 and 480 nm. The emission peak occurred at 420 nm at λexc 345 nm and there was a decrease in the fluorescence intensity by increasing the excitation wavelength. It is clear that the fluorescence emission of Arg CDs was significantly decreased for λexc 375 nm and disappeared for λexc 465 nm. However, significant fluorescence emission intensity was observed at λexc 520 nm in the range of 465–480 nm for Arg-FSS and Arg-RBF CDs, which was attributed to the emission from the modifying groups, FSS and RBF, on the Arg CDs, which is shown in Figure 4b,c. According to the literature, FSS has a maximum λ_em_ at 515 nm upon excitation at λ_exc_ 460 nm [36]; similarly, RBF fluorescence dye has a maximum λ_em_ at 520 nm for λ_exc_ 455 nm [23]. Therefore, it can be presumed that the modified groups of the Arg-FSS and Arg-RBF CDs provided green fluorescence properties with a maximum emission wavelength of 520 nm in addition to purple-blue fluorescence regions, thereby affording dual fluorescent properties. The QY values of these CDs were also calculated according to the quinine sulfate standard at 345 nm excitation and the fluorescence sodium salt (FSS) standard at 510 nm excitation. The QY values of Arg, Arg-FSS, and Arg-RBF CDs were found to be 12.5 ± 0.2%, 11.9 ± 2.7%, and 8.1 ± 0.9%, respectively, according to the quinine sulfate standard upon 345 nm excitation. On the other hand, the QY values of Arg-FSS and Arg-RBF CDs were determined to be 9.1 ± 2.0% and 23.2 ± 5.6%, respectively, according to the fluorescein dye standard upon 520 nm excitation. These results indicated that bare Arg CDs and Arg-FSS CDs had higher QY values at 345 nm excitation, but Arg-RBF CDs showed the highest QY value at 520 nm excitation. To assess the colloidal stability of these CDs, digital camera images of Arg, Arg-FSS, and Arg-RBF CD suspensions in DI water under UV light illumination at 366 nm after 1-, 3-, and 5-day wait times are demonstrated in Figure S1. It was clearly seen that all types of CDs were highly colloidally stable for up to 5 days. 

**Figure 5:**
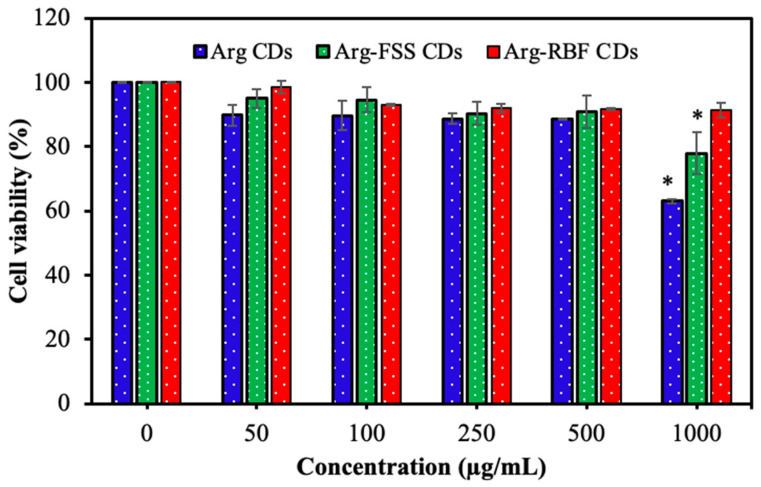
Cell viability of L929 fibroblast cells upon contacting Arg, Arg-FSS, and Arg-RBF CDs up to 1000 μg/mL concentration for 24 h incubation and the values are presented as mean ± SD, n = 3, * *p*-value < 0.05.

The cytotoxicity of the CDs was determined by investigating the cell viability of L929 fibroblast cells upon contacting the CDs at a concentration range of 50–1000 μg/mL for 24 h incubation, and the corresponding results are shown in Figure 5. 

As seen from Figure 5, the cell viability of the fibroblasts was not significantly affected by all Arg-based CDs up to 500 μg/mL concentration. Furthermore, Arg and Arg-FSS CDs induced lower cell viability at 1000 μg/mL concentration with 63.0 ± 6.5% and 77.8 ± 2.2% cell viability, respectively, in comparison to Arg-RBF CDs that induced 91.2 ± 0.7% cell viability at 1000 μg/mL. As reported in the literature, RBF has no important toxicity and is safely used as a food additive, food colorant, and supplement up to 35 mg per day for a 70 kg human [37]. Similarly, FSS is accepted as a non-toxic molecule and can be used as a diagnostic agent in clinical applications [30]. Therefore, upon conjugation onto Arg CDs, these agents somewhat improved the toxicity of bare Arg CDs, enhancing their biomedical application potential. 

**Figure 6:**
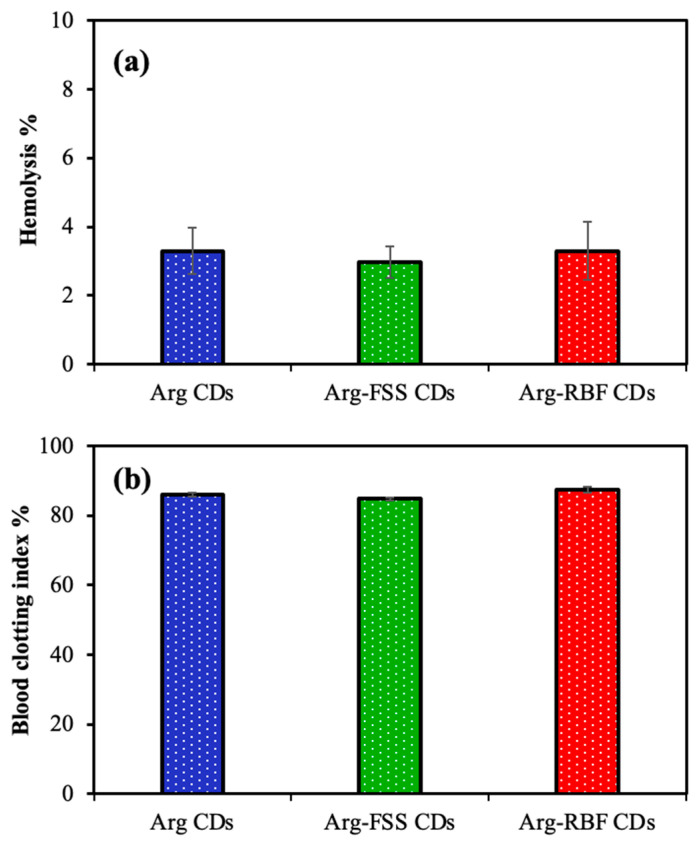
Hemocompatibility of Arg, Arg-FSS, and Arg-RBF CDs by (**a**) hemolysis and (**b**) blood clotting index assays at 1000 μg/mL concentration.

The hemocompatibility of Arg-based CDs was determined by hemolysis and blood clotting assays. As shown in Figure 6a, the hemolysis ratios (%) of Arg, Arg-FSS, and Arg-RBF CDs at 1000 μg/mL concentration were found to be 3.2 ± 0.6%, 2.9 ± 0.4%, and 3.3 ± 0.8%, respectively. It is well known that material in contact with blood can be accepted as hemocompatible if a 5% hemolysis ratio for red blood cells is obtained [38]. From these results, it can be reasoned that all types of CDs had similar toxic effects on the erythrocyte cells, with slight hemolysis ratios below 5%, and could be acknowledged as safe materials for intravenous applications. Furthermore, no important blood clotting effect was detected for 1000 μg/mL concentration of Arg, Arg-FSS, and Arg-RBF CDs, with 85.9 ± 2.5, 84.6 ± 5.0, and 87.5 ± 6.5 blood clotting index values, respectively, as shown in Figure 6b. Similar to these results, FSS and RBF molecules have been used intravenously as diagnostic agents and are widely accepted as safe [30,37]. All of these cytotoxicity and hemocompatibility results indicated that Arg CDs and modified forms could be used for biological applications up to 1000 μg/mL concentration. 

The antibacterial and antifungal potencies of the prepared CDs were analyzed by microtiter dilution assay against common microorganisms, e.g., *E. coli* and *K. pneumoniae* as Gram-negative bacteria, *S. aureus* and *B. subtilis* as Gram-positive bacteria, and *C. albicans* as a fungus. The minimum inhibitory concentration (MIC, mg/mL) values of the CDs are given in Table 1.

As seen from the antipathogenic test results, the MIC values of Arg CDs were found to be 3.12, 6.25, 3.12, 1.50, and 6.25 mg/mL against *E. coli*, *K. pneumoniae*, *S. aureus*, *B. subtilis*, and *C. albicans,* respectively. It is apparent that Arg CDs showed broad-spectrum antimicrobial activity against pathogenic bacterial and fungal species. The antibacterial and antifungal activities of Arg-based CDs were slightly decreased, e.g., almost two-fold compared to the increased MIC values for the modified Arg-FSS and Arg-RBF CDs. The zeta potential values of these CDs were analyzed to corroborate their antibacterial activity with surface charge, and zeta potential values of −5.4 ± 4.8, −15.8 ± 5.2, and −24.5 ± 10.2 mV were measured for Arg, Arg-FSS, and Arg-RBF CDs, respectively, at neutral pH conditions. These results showed that the highest antibacterial potency of the bare Arg CDs could be due to the relatively higher positive zeta potential value because of the presence of the related functional groups, e.g., -NH_2_, on their surface. It was found that Arg-FSS was most effective against *B. subtilis* bacteria. Therefore, overall, it could be assumed that these dye molecules, FSS and RBF, as modifying agents, did not strongly assert antimicrobial activity against these species and slightly inhibited the antimicrobial susceptibility to the modified forms of the Arg CDs; however, they improved the optical properties as well as the toxicity of the Arg CDs. 

**Figure 7:**
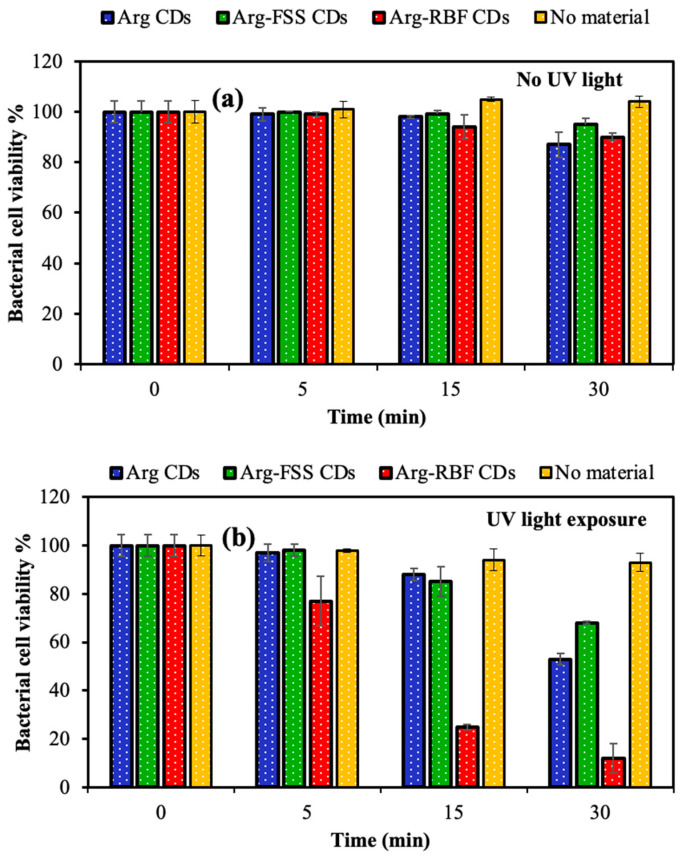
Bacterial cell viability (%) of *B. subtilis* in the presence of 1000 μg/mL concentration of Arg, Arg-FSS, and Arg-RBF CDs and without CDs under (**a**) no UV light and (**b**) UV light exposure for different incubation times.

The bacterial inhibition effects of N-doped CDs are generally dependent on the reactive oxygen species (ROS) activity of the CDs upon light exposure by means of their photo-induced properties in addition to their cationic structure [3,39,40,41]. The photodynamic antimicrobial inhibitory ability of 1000 μg/mL concentration of Arg, Arg-FSS, and Arg-RBF CDs was determined against *B. subtilis* upon UV light (315–400 nm, UVA, 300 W) exposure for 5, 15, and 30 min incubation times and compared with the catalytic performance under dark conditions. The corresponding results are demonstrated in Figure 7.

Under no UV light illumination, no significant bacterial inhibition was observed up to 30 min for each material, as shown in Figure 7a. However, inhibition of bacterial growth was slightly initiated in the presence of Arg-based CDs after 15 min UV light exposure and increased with almost half of bacterial killed after 30 min UV light exposure, as seen in Figure 7b. In addition, only UV light exposure without any CDs resulted in the absence of significant bacterial inhibition, with 93 ± 3.8% bacterial viability after 30 min irradiation. The highest photodynamic activity was measured for Arg-RBF CDs upon UV light exposure, with only 25 ± 1% and 12 ± 6% bacterial viability after 15 and 30 min, respectively. Thus, it was obvious that 30 min was enough time to eradicate *B. subtilis* using Arg-RBF CDs, but other CDs, such as Arg and Arg-FSS CDs, were not as effective for total inhibition of the pathogens up to 1000 μg/mL concentration under UV light irradiation. 

Some studies reported that RBF is a photo-activatable antibacterial material used in corneal ulcer treatment [29], in addition to its wide-spectrum antipathogenic activity upon UV light activation [22,24,25,26]. However, bare FSS has not shown antipathogenic activity [28]. It is well known that light-activated RBF oxidizes the guanine organic base in the DNA or RNA structure of pathogenic microorganisms, preventing replication of the pathogen’s genome and inhibiting pathogenic growth. Therefore, photo-induced RBF produces reactive oxygen species (ROS) via its fluorescence property, which can eradicate pathogens [24,25]. Similarly, some fluorescence CDs, such as Arg CDs, show light-activated antimicrobial properties depending on their high ROS generation capability and cationic structure [20]. Here, the photo-induced antibacterial activity of N-doped Arg CDs was improved by modification with the RBF molecule without invoking toxicity. 

## 4. Conclusions

In summary, fluorescein sodium salt (FSS) and riboflavin (RBF) fluorescence dyes were conjugated to Arg CDs to obtain modified Arg-FSS and Arg-FSS CDs for rapid microbial eradication by photodynamic therapy. The Arg-based CDs with a nanometer size range (<5 nm) and graphitic crystalline morphology were prepared and chemically modified with FSS and RBF. In addition to strong bright blue fluorescence emission at 420 nm under 345 nm excitation for Arg CDs, a slightly green fluorescence intensity at 520 nm was observed under 460 nm excitation for Arg-FSS and Arg-RBF CDs. It was uncovered that conjugation with dyes did not render any toxicity, and, in contrast, it improved the cytotoxicity of the modified Arg-based CDs at high concentration. Also, the presence of dyes on Arg CDs did not induce unwanted hematolytic behavior. Photodynamic antimicrobial activation against common pathogens was achieved using the modified Arg-RBF CDs under 30 min UV-A light exposure without any significant cytotoxicity and hemocompatibility, even at 1000 μg/mL concentration. Thus, modification of Arg CDs with RBF improved the potential use of this material, which demonstrated good biocompatibility and photodynamic antimicrobial activation owing to its rapid microbial killing capability upon UV-A light exposure. Overall, the prepared Arg-RBF CDs were found to possess intriguing antipathogenic effects through photodynamic activation in addition to great biosafety.

## Figures and Tables

**Table 1:** Antimicrobial effects of Arg, Arg-FSS, and Arg-RBF CDs by the minimum inhibitory concentration (MIC mg/mL) values against *E. coli*, *K. pneumoniae*, *S. aureus*, *B. subtilis*, and *C. albicans*.

Materials	Minimum Inhibitory Concentration (MIC, mg/mL)				
	*E. coli*	*K. pneumoniae*	*S. aureus*	*B. subtilis*	*C. albicans*
Arg CDs	3.12	6.25	3.12	1.50	6.25
Arg-FSS CDs	6.25	12.50	6.25	3.12	6.25
Arg-RBF CDs	6.25	12.50	6.25	6.25	6.25

## Data Availability

Data will be made available upon request.

## Supplementary Materials

The following supporting information can be downloaded at: https://www.mdpi.com/article/10.3390/ma16196512/s1, Figure S1: Digital camera images of Arg, Arg-FSS, and Arg-RBF CD suspensions under UV light illumination at 366 nm after 1-, 3-, and 5-day wait times.
