# Supplementary material for: Rapid Pathogen Purge by Photosensitive Arginine–Riboflavin Carbon Dots without Toxicity
Source: Materials (Basel). 2023 Sep 30;16(19):6512. doi: 10.3390/ma16196512 (PMC10573186; doi:10.3390/ma16196512)
Supplement: Supplementary file 1 [file materials-16-06512-s001.zip › materials-2628881-supplementary.pdf]

Rapid Pathogen Purge by Photosensitive Arginine-Riboflavin Carbon Dots without Toxicity

Selin S. Suner [1], Venkat R. Bhethanabotla [2], Ramesh S. Ayyala [3] and Nurettin Sahiner [1,2,3]*

[1] Department of Chemistry, Faculty of Sciences & Arts, and Nanoscience and Technology Research and Application Center (NANORAC), Canakkale Onsekiz Mart University Terzioglu Campus, Canakkale, 17100, TURKEY; sagbasselin@gmail.com; sahiner71@gmail.com; nsahiner@usf.edu

[2] Department of Chemical, Biological, and Materials Engineering, Materials Science and Engineering Program, University of South Florida, Tampa, FL 33620, USA; bhethana@usf.edu; sahiner71@gmail.com; nsahiner@usf.edu

[3] Department of Ophthalmology, Morsani College of Medicine, University of South Florida Eye Institute,12901 Bruce B Down Blvd, MDC 21, Tampa, FL 33612, USA; rsayyala@gmail.com (R.S.A.); sahiner71@gmail.com (N. S.)

* Correspondence: sahiner71@gmail.com ; nsahiner@usf.edu

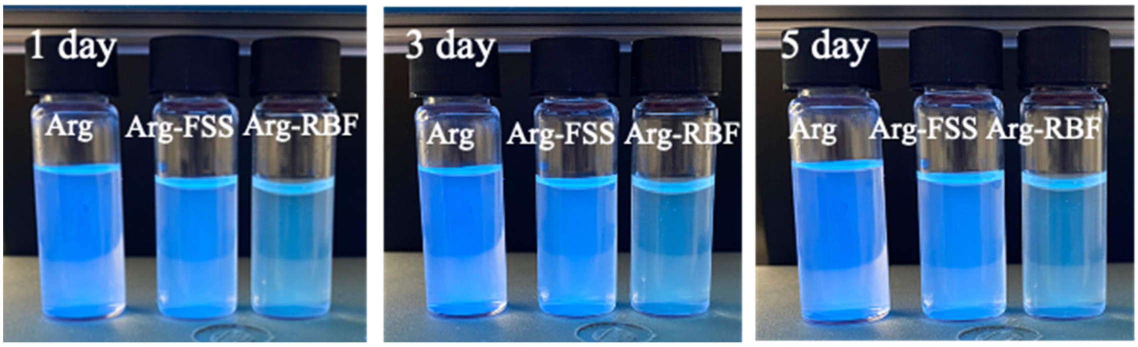

Figure S1. Digital camera images of Arg, Arg-FSS, and Arg-RBF CDs suspensions under UV light illumination at 366 nm wavelength after 1, 3, and 5 day wait times.
